# Extended spectrum β-lactamase producing uropathogenic *Escherichia coli* and the correlation of biofilm with antibiotics resistance in Nepal

**DOI:** 10.1186/s12941-019-0340-y

**Published:** 2019-12-17

**Authors:** Raju Shrestha, Santosh Khanal, Pramod Poudel, Karan Khadayat, Sajani Ghaju, Anita Bhandari, Sunil Lekhak, Narayan Dutt Pant, Manisha Sharma, Bishnu P. Marasini

**Affiliations:** 1 Department of Microbiology, National College, Tribhuvan University, Naya Bazar, Kathmandu, Nepal; 2 Department of Biotechnology, National College, Tribhuvan University, Naya Bazar, Kathmandu, Nepal; 3 Department of Microbiology, Goldengate International College, Tribhuvan University, Kathmandu, Nepal; 4 Department of Microbiology, Grande International Hospital, Kathmandu, Nepal

**Keywords:** *E. coli*, UTI, ESBL, Biofilm, *pgaA* and *pgaC*

## Abstract

**Background:**

Urinary tract infection (UTI) is one of the frequently diagnosed infectious diseases which is caused mainly by *Escherichia coli*. *E. coli* confers resistance against the two major classes of antibiotics due to the production of extended spectrum β-lactamase enzymes (ESBL), biofilm, etc. Biofilm produced by uropathogenic *E. coli* (UPEC) protects from host immune system and prevent entry of antimicrobial compounds. The main objective of this cross-sectional study was to determine the correlation of biofilm production and antibiotic resistance as well as to characterize the *pgaA* and *pgaC* genes responsible for biofilm formation among uropathogenic ESBL producing *E. coli.*

**Methods:**

A total of 1977 mid-stream urine samples were examined and cultured for bacterial strain identification. ESBL was detected by combined disc method following CLSI whereas biofilm formation was analyzed by semi-quantitative method. Furthermore, the *pgaA* and *pgaC* genes responsible for biofilm formation in UPEC were detected by multiplex PCR. All the statistical analyses were done via IBM SPSS Statistics 21 where Pearson’s correlation test were used to determine correlation (−1 ≥ *r *≤ 1).

**Results:**

*E. coli* was the predominant causative agent, which accounted 159 (59.3%) of the Gram-negative bacteria, where 81 (50.9%) *E. coli* strains were found to be ESBL producers. In addition, 86 (54.1%) *E. coli* strains were found to be biofilm producers. Both the *pgaA* and *pgaC* genes were detected in 45 (93.7%) the UPEC isolates, which were both biofilm and ESBL producers. Moreover, there was a positive correlation between biofilm and ESBL production.

**Conclusion:**

The analyses presented weak positive correlation between biofilm and ESBL production in which biofilm producing UPEC harbors both *pgaA* and *pgaC* genes responsible for biofilm formation.

## Background

Over 150 million new cases of urinary tract infection (UTI) are diagnosed worldwidely per year [1, 2], therefore, UTI is one of the health complications that need a serious concern [3]. *Escherichia coli* are the main causative agent of UTI fulminating prostatitis, biliary tract infection, and urinary catheter cystitis [4] which accounts approximately 80 to 85% of the cases [5–7]. Biofilm (poly-β-1,6-*N*-acteyl-d-glucosamine i.e., PGA) production is one of the arsenals of *E. coli* to invade the host. The *pgaABCD* locus of *E. coli* is required for synthesis of biofilm and other pathogenic role [4, 17]. The biofilm formation via *pgaABCD* depends on various factors viz. fimbriae, type I pili, motility, etc. This class of polysaccharides in *E. coli* was recently discovered and acts as an adhesive in biofilms [4]. Biofilms help not only in the transfer of plasmid encoding resistance genes i.e., ESBL to other organisms via conjugation but also resist immune clearance [9–13]. The dissemination of ESBLs has emerged to a high proportion of CTX-M enzymes, notably *E. coli,* which is the major carriers of ESBL-encoding genes i.e. *bla*_CTX-M_ [11, 14, 15] so, the incidence of ESBL producing *E. coli* is now elevating in urinary tract infections [16].

The uropathogenic *E. coli* is now developing new trends of antimicrobial resistance as well as their biofilm is supporting to gain the resistance against numerous antibiotics [6, 8, 13]. To our knowledge, this study would be first in Nepal to determine the correlation of biofilm formation and antibiotic resistance as well as to characterize the biofilm producing genes located in *pgaABCD* locus among uropathogenic ESBL producing *E. coli.*

## Methods

The cross-sectional study was carried out in the Department of Microbiology, Grande International Hospital, Tokha, Kathmandu, and Department of Microbiology, National College, Kathmandu from June to November, 2017. A clinical and socio-demographic study of patients was performed. A total of 1977 mid-stream urine were cultured semi-quantitatively on Cysteine Lactose Electrolyte Deficient Agar plates and incubated at 37 °C for 24 h [6, 18, 19]. The antibiotic susceptibility test was performed by modified Kirby–Bauer method of disk diffusion within the guidelines of Clinical and Laboratory Standard Institute (CLSI), 2015 [18–20].

### Detection of ESBL producing uropathogenic *E. coli*

The resistance of cefotaxime (30 µg) in *E. coli* was used as the screening method for detection of ESBL which were then confirmed by combined disc method following CLSI, 2015 [20].

### Detection of biofilm production in *E. coli*

The uropathogenic *E.coli* were cultured in 5 ml of Luria–Bertani (LB) broth at 37 °C for 24 h. The turbidity of cultured LB broth was compared with the 0.5 McFarland standard to maintain 10^8^ CFU/ml followed by addition of LB broth supplemented with 1% glucose in the ratio 1:100 to maintain the concentration of approximately 10^6^ CFU/ml. It was then vortexed and 200 μl of diluted cultured LB broth was transferred per well in a microtiter plate in triplicate. A positive control i.e. 200 μl of *E. coli* ATCC 25922 cultured LB broth and a negative control i.e. 200 μl of LB broth were transferred into well of a microtiter plate in triplicate. The microtiter plates were covered with a tape and incubated at 37 °C for overnight. The plates were washed 3 times with 300 μl of sterile phosphate buffered saline (PBS, pH 7.2). Subsequently, plates were heat fixed by incubating at 60 °C for 1 h. Then, the plates were stained with 150 μl of 2% crystal violet for 15 min at room temperature. The plates were washed with distilled water until the stain was free. It was then air dried at room temperature. Afterward, 150 μl of 95% ethanol (v/v) was transferred per well in microtiter plates. The covered microtiter plates were left at room temperature for half an hour without shaking. The absorbance was measured at 570 nm using a spectrophotometer. The uropathogenic *E. coli* was classified as a non-biofilm producer, weak biofilm producer, moderate biofilm producer, or strong biofilm producer on the basis of findings evaluated [21, 22].

### Detection of biofilm genes i.e. *pgaA and pgaC* in *E. coli*

The genomic DNA was extracted from the ESBL and biofilm producing uropathogenic *E. coli* via a standard phenol–chloroform protocol [23]. Multiplex PCR was done to detect *pgaA* and *pgaC* genes in which the *pgaA* and *pgaC* primers were used for the amplification of 209 and 540 bp, respectively (Table 1) [24, 25].

**Table 1:** The forward and reverse primers used in *pgaA* and *pgaC* genes

Gene	Primers	Sequences	GC %	Tm (°C)
*pgaA*	Forward	5′-GGCTTTGAAACTTCTTACTGC-3′	42.9	57.4
	Reverse	5′-CCTGTTTATCTTGCCCGGCC-3′	60	62.5
*pgaC*	Forward	5′-ATGATTAATCGCATCGTATCG-3′	38.1	55.5
	Reverse	5′-CATCGGTTCCACAATATATGC-3′	42.9	57.4

At first, 12.5 µl of Master Mix (Biolabs, New England) was added followed by 8.5 μl nuclease-free water, 0.5 µl of each primer (Macrogen, Inc., South Korea) of both genes and 2 µl of DNA from the bacterial strains to maintain 25 μl PCR mixture (TAKARA PCR Thermal Cycler Dice Gradient TP600, Takara bio, Tokyo, Japan). PCR conditions i.e., initial 5 min denaturation step at 94 °C was maintained followed by 32 cycles of 30 s at 94 °C, 30 s at 50 °C, and 45 s at 72 °C, and a final extension step of 5 min at 72 °C [24].

### Data analysis

All the data collected were analyzed via IBM SPSS Statistics 21. Pearson’s correlation test were used to determine correlation (−1 ≥ *r *≤ 1) [1, 6].

## Results

Among 1977 mid-stream urine samples, a total of 311 (15.7%) isolates were isolated with significant growth i.e., ≥ 10^5^ cfu/ml where 159 (51.1%) *E. coli* strains were isolated. Out of 159 *E. coli* strains, 81 (50.9%) were ESBL producer, 86 (54.1%) were biofilm producer in which 48 (30.2%) were both ESBL and biofilm producer. Within 48 *E. coli* strains, which were both ESBL and biofilm producer, 45 (93.7%) strains showed both *pgaA* and *pgaC* genes that are responsible for biofilm production.

### Clinical and socio-demographic study

Community-acquired infections was found to be higher which accounts 218 (70.1%) and female were affected by 173 (55.3%). Moreover, the higher number of cases was observed within the age group 60+ which accounts 93 (29.91%) (Table 2).

**Table 2:** Clinical and socio-demographic study

S. No.	Status of patient	Number (%)
1	In-patient	93 (29.9%)
2	Out-patient	218 (70.1%)

S. No.	Gender	Number (%)
1	Male	138 (44.7%)
2	Female	173 (55.3%)

S. No.	Age distribution	Number (%)
1	0–20	28 (9%)
2	20–40	99 (31.83%)
3	40–60	91 (29.26%)
4	60+	93 (29.91%)

### Antibiotics susceptibility profile

Out of 159 *E. coli* strains, 82 (51.6%) and 90 (56.6%) were resistant towards to cefotaxime and cotrimoxazole, respectively whereas 159 (100%) *E. coli* strains were sensitive towards tigecycline (Table 3).

**Table 3:** Antibiotic susceptibility profile of uropathogenic *E. coli* isolates

Antibiotic used	Sensitive	Intermediate	Resistance	Total isolates
Amoxyclav	65 (40.9%)	12 (7.5%)	82 (51.6%)	159
Cefotaxime	69 (43.4%)	8 (5%)	82 (51.6%)	159
Colistin	140 (88.1%)	0 (0%)	19 (11.9%)	159
Cotrimoxazole	69 (43.4%)	0 (0%)	90 (56.6%)	159
Gentamycin	124 (78%)	9 (5.7%)	26 (16.4%)	159
Meropenem	121 (76.1%)	24 (15.1%)	14 (8.8%)	159
Nitrofurantoin	135 (84.9%)	14 (8.8%)	10 (6.3%)	159
Norfloxacin	52 (32.7%)	8 (5%)	99 (62.3%)	159
Tigecycline	159 (100%)	0 (0%)	0 (0%)	159

### Detection of biofilm formation by the semi-quantitative method

**Fig. 1:**
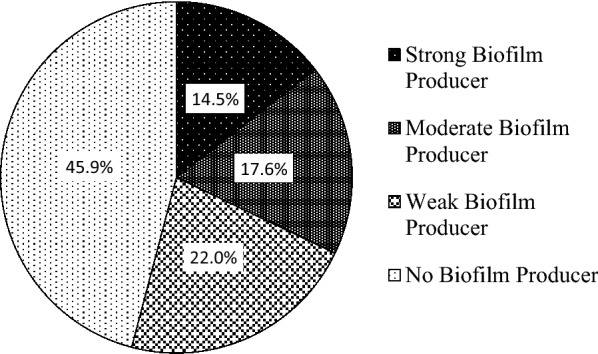
Detection of biofilm production in uropathogenic *E. coli* via semi-quantitative method

Out of 159 (51.1%) UPEC, 23 (14.5%) strains were found to be strong biofilm producer, 28 (17.6%) strains were moderate biofilm producer and 35 (22%) strains were weak biofilm producer whereas 73 (45.9%) strains were found to be biofilm non-producer (Fig. 1).

### Correlation between biofilm production by the semi-quantitative method and ESBL production in *E. coli.*

Amongst ESBL producing UPEC, 18.5%, 17.3%, and 23.5% showed strong, moderate and weak production of biofilm, respectively. There was a weak positive correlation between biofilm formation and ESBL production (*r *= 0.157) which is illustrated in Table  4.

**Table 4:** Correlation between biofilm and ESBL production in *E. coli*

ESBL detection	Strong biofilm producer	Moderate biofilm producer	Weak biofilm producer	No biofilm producer	Total isolates	*R*-value
ESBL Producer	15 (18.5%)	14 (17.3%)	19 (23.5%)	33 (40.7%)	81 (50.9%)	0.157
ESBL non-producer	8 (10.3%)	14 (17.9%)	16 (20.5%)	40 (51.3%)	78 (49.1%)	
Total	23 (14.5%)	28 (17.6%)	35 (22%)	73 (45.9%)	159 (100%)	

### Detection of biofilm genes i.e., *pgaA and pgaC* in *E. coli*

**Fig. 2:**
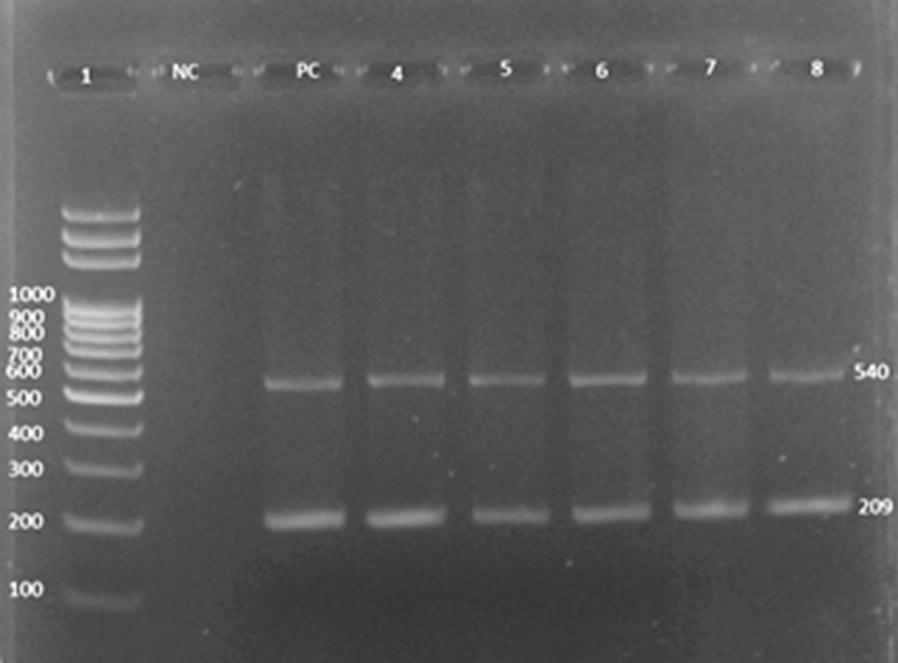
The *pgaA* and *pgaC*genes detection in *E. coli* strains: lane 1 is the DNA ladder labeling from 100 to 1000 bp, NC and PC are negative and positive controls, respectively; lanes 4, 5, 6, 7 and 8 are positive for both the *pgaA* and *pgaC*genes at 209 bp and 540 bp, respectively

Among 48 uropathogenic *E. coli* processed which were ESBL and biofilm producers, 14 (93.3%) strong, 14 (100%) moderate and 17 (89.5%) weak biofilm producing and ESBL producing UPEC were found to contain both *pgaA* and *pgaC* genes which is amplified at 209 bp and 540 bp, respectively (Fig. 2). *E. coli* ATCC 25922 was used as a positive control for *pga*genes (Table 5).

**Table 5:** Detection of *pgaA* and *pgaC* genes by multiplex PCR

Sample	Detection of *pgaA* and *pgaC* genes		Total
	Positive	Negative	
Strong biofilm producer	14 (93.3%)	1 (6.7%)	15
Moderate biofilm producer	14 (100%)	0	14
Weak biofilm producer	17 (89.5%)	2 (89.5%)	19
Total	45 (93.7%)	3 (6.3%)	48

## Discussion

Urinary tract infections are frequently occurred infections in hospital where 93 (29.9%) were hospital acquired infections. The prevalence rate of urinary tract infections in female was found to be predominant (55.3%) than male (44.7%) because of the close proximity between vagina and anus [1], cystitis, sexual behavior, vaginal infections, pregnancy, diabetes mellitus, obesity and genetic sensitivity in female [2, 36]. In addition, the prevalence rate of infection was found to be higher in age groups 60+ years. From the above evidences, it was clear that urinary tract infections were found to be more prone to older ages rather than younger ages. It is due to the fact that with the ageing, immune response tends to decline gradually and also hormonal changes takes place which leads to infections of urinary tract [36].

*E. coli* was found to be a predominant causative agent of UTI which was highly resistant towards norfloxacin 99 (62.3%), cotrimoxazole 90 (56.6%) and cefotaxime 82 (51.6%) and their resistance patterns were found to be similar with the earlier study conducted [8, 10, 11]. There was a weak positive correlation (*r *= 0.157) relationship between biofilm production and ESBL productions. Within strong, moderate and weak biofilm producing *E. coli,* 65.2%, 50% and 54.3% were ESBL producer, respectively. There was positive correlation between biofilm and ESBL producing *E. coli* which was stated by Tabasi et al. and Neupane et al. [6, 8]. This revealed that biofilm favors the ESBL gene transferred between the *E. coli* and other microorganisms because of matrix which stabilizes and enhances the transferability of genetic elements horizontally as well as resist the immune clearance [6, 21, 30–32].

The *pgaABCD* locus is selected to detect the *pgaA* and *pgaC* genes by multiplex PCR as it contributes in production of β-1,6-*N*-acetyl-d-glucosamine, surface adherence as well as intracellular adhesion [24, 25]. The protein, PgaC is responsible for production of β-1,6-*N*-acetyl-d-glucosamine as it utilizes UDP-*N*-acetyl glucosamine as a substrate, and PgaA helps in translocation and anchoring of β-1,6-*N*-acetyl-d-glucosamine to cell surfaces [4]. The *pgaA* and *pgaC* genes were found to harbor in 45 (93.7%) out of 48 biofilm as well as ESBL producing UPEC. Nonetheless, *pga* locus was found to be absent in 3 (6.3%) biofilm as well as ESBL producing UPEC. It may be due to involvement of variety of genes, i.e. *crl, csg, cvaC, fimA, fimH, iutA, ompC, ompF, sfaS, traT, yidC,* etc. responsible for the production of biofilm [24, 34, 35].

The development of resistance in *E. coli* may be due to haphazard use of antibiotics, plasmid-mediated genes, i.e*. bla*_CTX-M_*, bla*_SHV_*, bla*_OXA_, etc., quorum sensing, etc. [26–29]. The rise of multidrug-resistant UPEC poses a serious threat to manage UTI along with increment in treatment cost. The biofilm producing pathogens are sensitive towards co-therapy with macrolides i.e. erythromycin, clarithromycin and azithromycin, and other effective antibiotics as macrolides are considered as reliable anti-biofilm agents [6, 33].

## Conclusion

In conclusion, tigecycline were found to be pragmatic approach for treatment as the result indicates in this research. There was found to be weak positive correlation between biofilm and ESBL production. In addition, biofilm producing UPEC harbors both *pgaA* and *pgaC* genes responsible for biofilm production.

### Limitations of study

The study of all genes responsible for biofilm production other than *pgaA* and *pgaC* genes and the genes ESBL productions could not be carried out. Genes like mcr-1 and NDM-1 for colistin and meropenem resistant strains were not performed respectively to confirm the resistivity.

## Data Availability

Data sharing is not applicable to this paper as the datasets generated needed to be confidential.

## Abbreviations

CLSI: Clinical and Laboratory Standards Institute
UPEC: uropathogenic *E. coli*
